# Donor gonadal vein reconstruction for extension of the transected renal vessels in living renal transplantation

**DOI:** 10.4103/0970-1591.65417

**Published:** 2010

**Authors:** Muthu Veeramani, Vikas Jain, Arvind Ganpule, R. B. Sabnis, Mahesh R. Desai

**Affiliations:** Department of Urology, Muljibhai Patel Urological Hospital, Dr. V V Desai Road, Nadiad, Gujarat - 387 001, India

**Keywords:** Donor gonadal vein, laparoscopic donor nephrectomy, renal transplantation

## Abstract

**Introduction:**

Donor gonadal vein is a readily available vascular reconstruction material for vascular reconstruction, for difficult situations, in living related renal transplantation. Vein extension with the gonadal vein has been described as a simple and safe method to elongate renal vein especially in right living donor kidneys. We applied the donor gonadal vein for lacerated accessory renal artery and renal vein reconstruction.

**Materials and Methods:**

The donor gonadal vein was used to reconstruct the lacerated accessory renal artery in one patient. The donor gonadal vein was isolated, used as an interposition graft to bridge the gap between transected accessory renal artery and external iliac artery of the recipient. In another patient, gonadal vein was used to reconstruct short right renal vein, which got damaged during retrieval.

**Results:**

This technique resulted in a tension-free anastomosis. There were no procedure related complications. The ischemia time remained within acceptable limits and grafts showed excellent outcomes.

**Conclusions:**

The use of gonadal vein for renal vascular reconstruction seems to be an acceptable option during living related renal transplantation, lest the need arise, with no increased graft morbidity.

## INTRODUCTION

Laparoscopic donor nephrectomy (LDN) has become the standard of care for graft retrieval in living renal transplant situations. In addition to minimizing donor morbidity, hospital stay, and convalescence, LDN has also been shown to provide equivalent short- and long-term renal allograft functional outcomes compared with open surgery.[1,2] With increasing experience, LDN has been extended to donors with multiple renal arteries with no adverse impact on long term outcome as compared with those with single renal arteries.[3] However, a meticulous handling of hilar structures, based on pre-operative angiographic findings, is a pre-requisite for avoiding intra-operative vascular trauma and successful graft procurement. Intra-operative trauma to accessory renal vasculature is known to occur and poses problems in vascular reconstruction at the recipient side. Injury and laceration to accessory arteries is one such trauma which leaves the transplant surgeon with limited options of vascular reconstruction, especially when it involves a large segment and results in arterial segment of insufficient length to accomplish tension-free anastomosis. We utilized donor gonadal vein, in two such reconstructions, to lengthen the accidentally transected small lower accessory renal artery and short damaged right renal vein, respectively of living donor grafts.

## CASE REPORTS

### Case 1

**Figure 1:**
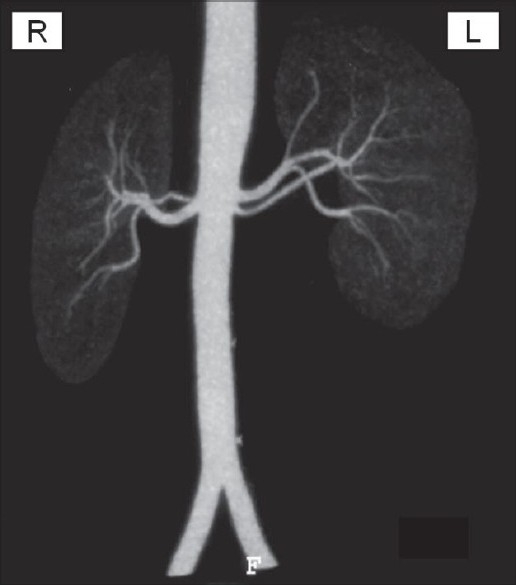
Donor’s renal angiography showing bilateral dual renal arteries. The upper and lower renal arteries, on the left side, measure 4.2 mm and 2.9 mm, respectively, at origins and the distance between two was 1.7 mm

A 49-year-old male with ADPKD and ESRD underwent living, right iliac fossa renal transplant on 25/01/2007. The donor had two renal arteries on either side [Figure 1]. Left kidney was harvested using standard transperitoneal laparoscopic approach. During retrieval, the lower smaller renal artery was accidentally cut and transected. The graft was harvested, in no time, through pfannensteil incision.

**Figure 2:**
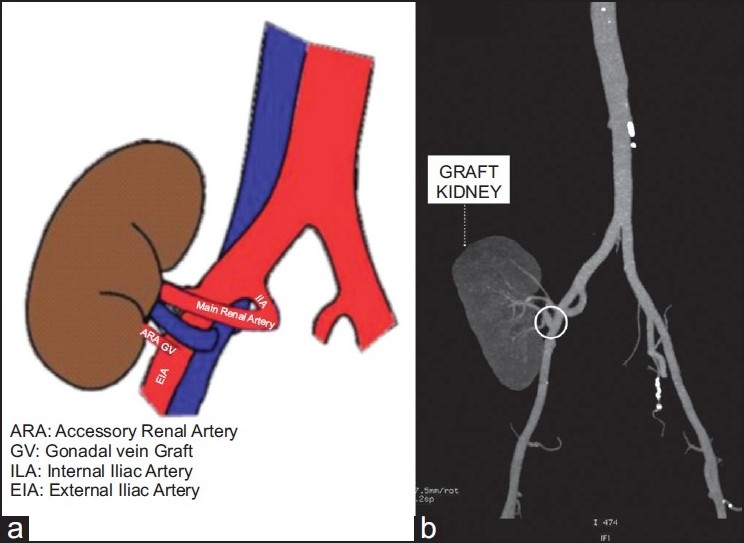
(a) Diagrammatic representation of the vascular reconstruction, showing gonadal vein interposition graft; (b) CT angiography, two years after the transplant, showing patent gonadal vein graft

Vascular reconstruction involved dual anastomosis, wherein the main renal artery was anastomosed, in an end-to-end manner, to the internal iliac artery (IIA) and the smaller renal artery was anastomosed to the external iliac artery (EIA) in an end-to-side manner. The transected renal artery was of insufficient length to accomplish a tensionfree anastomosis, with EIA, so a small segment of donor gonadal vein was dissected free of the adipose tissue and clips were removed. It was, then, used as an interposition graft to bridge the gap between the cut artery and the external iliac artery [Figure 2a]. The total ischemia time was 58 minutes. Patient made a remarkable recovery without any complications.

### Case 2

**Figure 3:**
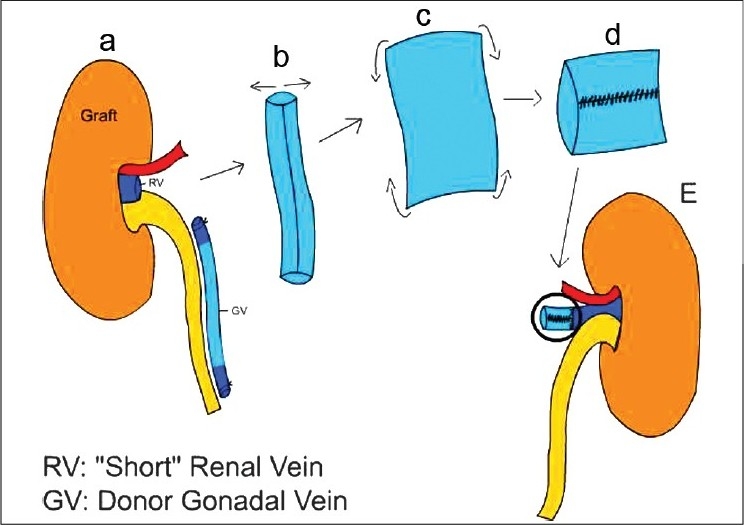
(a) Diagrammatic representation of the conduit preparation in second patient: Graft with “short” renal vein; (b) the gonadal vein was dissected free; (c) and opened longitudinally; (d) The lateral ends were sutured to make a conduit; (e) one end of which is sutured to the graft vein to lengthen it

A 50-year-old male with ESRD underwent living, right iliac fossa renal transplant in October, 2008. The donor had two renal arteries on the left side and single renal artery on the right side. Right kidney was procured by trans-umbilical, transperitoneal single port laparoscopic approach. During retrieval, through umbilical incision, the already small renal vein got damaged. Donor gonadal vein was, then, used to lengthen the graft vein. It was opened longitudinally and lateral walls were sutured with prolene 6-0 to make conduit [Figure 3]. One end of this conduit was sutured to the graft vein and the other end was sutured to the external iliac vein. There was no difficulty encountered during anastomosis. The total ischemia time was 82 minutes. There was smooth postoperative recovery with good graft function.

## RESULTS

Two years post transplant, the first patient maintains good graft function. His serum creatinine level and glomerular filtration rate, in Jan 2009, are 1.0 mg/dl and 108 ml/min respectively. His CT angiography shows patent gonadal vein graft [Figure 2b]. The second patient, at his last follow-up, eight-months after surgery, maintains as serum creatinine of 1.1 mg/dl and glomerular filtration rate of 88 ml/min.

## DISCUSSION

The incidence of intra-operative renal vascular injuries, during laparoscopic graft retrieval, is small in experienced hands. In one series[4] it was reported to be 7.7%. Since the arterial blood supply of the kidney is segmental, loss of arterial flow in the distribution of an accessory polar vessel can result in a significant area of infarction. This may progress to segmental necrosis of the graft and the devastating development of a urinary fistula. Therefore, the establishment of arterial blood flow to the distribution of this accessory polar vessel is mandatory.

The approach to a transected accessory polar artery is indicated by the point of transection, available length and diameter of the vessel. If the point of transection results in an arterial segment of sufficient length, the severed end of the artery may be anastomosed to the side of main renal artery, or to the side of the external iliac artery and also in an end-to-end manner to the inferior epigastric artery. However, an insufficient length of the transected artery, limiting any of the aforementioned anastomoses, poses a real challenge to the vascular reconstruction. Anatomic material like donor gonadal vein, can be used to facilitate graft implantation and achieve a tension-free vascularization at this critical point. Although the use of autogenous vein grafting in vascular surgery is routine, the use of an allogenic vein graft poses unique considerations in transplant setting. Of paramount importance is the durability of a small diameter allogenic vein and its possible rejection and subsequent thrombosis. The immunogenic mechanism involving the attachment of recipient antibodies to donor endothelium with subsequent complement activation, rapid fibrin deposition, and vessel occlusion has been described in renal transplantation.[5] Fear of this immunogenic response to allogenic “anatomic material” has compelled vascular surgeons to use autologous material like greater saphenous vein from the recipient, as arterial conduit to ensure tension-free anastomosis.[6] However, this requires an extra incision. The use of large diameter allogenic vascular conduits, both arterial and venous iliac vessel segments, to revascularize hepatic allografts has been described without subsequent immunologic occlusions.[7] Immunosuppression also, probably, spares allogenic vascular conduits from immunologic damage. The other possible option in this scenario is the use of synthetic or bank vascular grafts, which are associated with high risk of thrombosis.

The use of gonadal veins for vascular reconstruction and extension of the renal vein have been previously described.[8–10] The advantage of this strategy is the use of donor vascular tissue without donor or recipient hazard. In addition, the gonadal vein can easily be obtained during kidney procurement. The vein extension procedure can easily be performed and does not significantly extend the ischemia time.

The utilization of “small caliber” gonadal vein graft for arterial reconstruction was first reported by Hakaim *et al*.,[11] They used the gonadal vein interposition grafts to replace distally transected superior polar arteries in cadaveric renal transplantation. Ohwada *et al*.,[12] also used gonadal vein graft for hepatic artery reconstruction with no reported graft related complications.

## CONCLUSIONS

Atraumatic graft procurement is of critical importance in renal transplantation. Careful dissection and identification of anomalous vasculature is required for optimal success. All vascular remnants removed from the donor should be properly handled and preserved in iced slush to be able to exercise all options of organ revascularization. In this way they are in optimal condition for potential use during transplantation procedure, lest the need arise. The gonadal vein has an ideal diameter to accomplish renal vein as well as arterial reconstruction. The gonadal vein graft will be a new and preferable addition to the selection of an optimal graft for renal arterial reconstruction.
